# Management protocol and surgical techniques for MRI–Negative Cushing’s disease: a series of 6 cases

**DOI:** 10.3171/2023.4.FOCVID2318

**Published:** 2023-07-01

**Authors:** Nidhisha Sadhwani, Ashish Suri, Amol Raheja, Santanu Kumar Bora, Rajesh Khadgawat, Mehar Chand Sharma, Ajay Garg, Rajni Sharma

**Affiliations:** Departments of 1 Neurosurgery,; 2 Endocrinology,; 3 Neuropathology,; 4 Neuroradiology, and; 5 Paediatric Endocrinology, All India Institute of Medical Sciences, New Delhi, India

**Keywords:** Cushing’s disease, MRI negative, endoscopic transsphenoidal surgery, pituitary exploration, pituitary perfusion, pituitary surgery

## Abstract

Up to 40% of Cushing’s disease (CD) patients show no evidence of an adenoma on dynamic contrast-enhanced MRI. Inferior petrosal sinus sampling (IPSS) remains the gold standard for diagnosis in these patients. Remission rates in MRI–Negative CD are far less at 50%–71%, compared with patients in whom an adenoma is identified on MRI. Endoscopic endonasal transsphenoidal surgery is the surgical approach of choice in these cases. Various adjuncts can be used to localize an adenoma. In this video, the authors highlight their additional usage of pituitary perfusion MRI for identification of the adenoma. They present their stepwise management algorithm and surgical techniques for sellar and suprasellar exploration in 6 cases of MRI–Negative CD operated on by the senior author (A.S.).

The video can be found here: https://stream.cadmore.media/r10.3171/2023.4.FOCVID2318

## Transcript

In this video, we describe the management protocol and surgical techniques of exploration in 6 cases of MRI–Negative Cushing’s disease operated at our institute. Up to 40% of Cushing’s disease patients show no evidence of an adenoma on dynamic contrast-enhanced MRI.^1^ Gold standard for diagnosis in these patients remains inferior petrosal sinus sampling.^2^ Remission rates in MRI–Negative CD are far less—ranging from 50% to 71%;^3^ adequate initial treatment is thus important. The surgical approach of choice in these patients remains endoscopic endonasal transsphenoidal surgery.^4^ Various adjuncts can be used to localize an adenoma. In this video, we highlight our additional usage of pituitary perfusion MRI^5–10^ for identification of the adenoma. It utilizes the differential enhancement pattern of the normal pituitary gland, which is more avidly contrast-enhancing than the adenoma due to the differences in the vasculature of the normal and the adenomatous pituitary tissue. Pituitary perfusion curves thus help us identify the small adenomas which might be missed on routine dynamic MRI of the sella.

### 1:06 Case 1 Preoperative.

Our first patient is a 13-year-old female, a case of primary MRI–Negative Cushing’s disease with central gradient on IPSS. There was subtle thickening of the stalk on dynamic contrast-enhanced MRI of the sella, but there was no definite evidence of an adenoma. She underwent endoscopic endonasal transsphenoidal surgery with exploration of the pituitary gland. The patient positioning was as per the standard position used for an EETS, as shown here.

### 1:31 Case 1 Operative Procedure.

A binostril approach was used. Due to the narrow surgical corridor in this pediatric patient, a middle turbinectomy was performed through the right nostril. A posterior septostomy was made. The sphenoid rostrum was drilled and nibbled and the sphenoid sinus was entered. Similar procedure was repeated through the left nostril. The sellar floor was drilled and eggshelled. Dura was opened from 5-o’clock to 7-o’clock position and the gland was explored. Suspicious adenomatous tissue was seen in the right side of the gland. It was curetted out and sent for histopathological analysis.

### 2:22 Case 1 Postoperative.

Postoperatively, this patient had clinical and biochemical persistence of her disease, and histopathological examination did not reveal an adenoma. Thus, the patient underwent reexploration of the gland 2.5 months later.

### 2:35 Case 1 Redo Surgery.

Repeat imaging revealed subtle thickening of the stalk without any evidence of an adenoma. Thus, a suprasellar exploration was performed via an extended endoscopic endonasal transsphenoidal approach. The tuberculum sellae and planum sphenoidale were drilled. There was no evidence of an adenoma on exploration of the gland. The dura over the planum was cut and the pituitary stalk was exposed. Adenoma was identified on the anterior pituitary stalk and was curetted out. Normal pituitary stalk and the chiasm were seen. Closure was performed using fascia lata, fat and augmented with fibrin glue, as there was intraop CSF leak.

### 3:21 Case 1 Postoperative Course.

A lumbar drain was inserted in the immediate postoperative period as there was evidence of an intraop CSF leak. Nasal packs were removed after 48 hours. Lumbar drain was removed on postoperative day 5. There was no evidence of a CSF leak after the lumbar drain removal. The patient underwent clinical and biochemical remission, and histopathological examination revealed the presence of an adenoma.

### 3:42 Case 2.

Our second case is a 12-year-old male with primary MRI–Negative Cushing’s disease. IPSS revealed a central gradient with right-sided lateralization. This patient underwent surgery through the standard endoscopic approach. There was conchal type of pneumatization seen in this patient, as evident on the preoperative CT. Intraoperatively, the identification of sella was difficult due to the conchal type of pneumatization. Moreover, the neuronavigation was malfunctioning, and thus we were not able to precisely identify and confirm the intraoperative anatomy. Extra bony drilling had to be performed to obtain adequate exposure. Bone was drilled to identify the sellar floor. Dura was identified and opened. What appeared to be the sellar floor actually was the planum dura in proximity to the tuberculum sellae, as confirmed after the dural opening and arachnoid bulge. Thus, a suprasellar exposure was not intended and was performed by mistake. Further drilling was performed inferiorly and the sellar floor could then be located. The sellar dura was opened. Adenomatous tissue was identified on the right side of the gland. It was dissected out. The patient underwent clinical and biochemical remission, but no adenoma was identified on histopathological examination, as it might have been aspirated out due to its small size.

### 5:15 Case 3.

Our third patient is a 31-year-old female, a case of primary MRI–Negative Cushing’s disease with central gradient on IPSS. There was suspicious lesion at the junction of the upper end of the pituitary gland and lower end of the stalk on PET-CT and perfusion MRI. Surgical exploration was planned after several rounds of neurosurgery-neuroradiology-neuroendocrine tumor board meetings. She underwent exploration via an extended approach. The sellar floor, turberculum sellae, and the planum sphenoidale were drilled. Dura was opened. We first explored the upper middle part of the pituitary gland but did not find any adenoma. The pituitary stalk was then exposed, but we still did not find any tumor. The diaphragma was cut to expose the junction of the pituitary gland with the stalk. Adenoma was identified at the junction of the gland and the stalk and it was removed. A standard three-layered closure was performed with fat and augmented with glue, as there was intraop CSF leak. The patient underwent remission, and an adenoma was identified on histopathological examination.

### 6:27 Case 4.

Our fourth patient is a 29-year-old female, a case of recurrent MRI–Negative Cushing’s disease, operated twice before. She underwent her first surgery at another center, and the second surgery was performed at our center. She underwent remission after the previous two surgeries, and adenoma was confirmed on histopathology with an MIB index of 5%. She underwent reexploration of the gland. Dura was opened. A lateral sellar exploration was performed on both sides and suspicious adenomatous tissue was removed. Sella was explored. No adenoma was identified in the sella, and thus a hemihypophysectomy was performed. Sella was packed with fat and augmented with glue as there was suspicion of an intraop CSF leak. She did not undergo remission, and histopathology did not reveal an adenoma. She was thus managed with a bilateral adrenalectomy.

### 7:29 Case 5.

Our fifth patient is a 39-year-old female, a case of primary MRI–Negative Cushing’s disease. On pituitary perfusion MRI there was a suspicious lesion in the floor on the right side. She underwent exploration of the gland via a standard endoscopic endonasal transsphenoidal approach. Dura was opened from 5-o’clock to 7-o’clock position. Adenomatous tissue was seen in the right side of the gland which was removed. Lateral sellar exploration was performed and suspicious tissue was removed. Sella was packed with fat and augmented with glue as there was suspicion of an intraop CSF leak. The patient underwent delayed remission, although histopathological examination did not reveal an adenoma.

### 8:21 Case 6.

Our sixth patient is a 45-year-old female, a case of recurrent MRI–Negative Cushing’s disease, operated once before at a different center. She had undergone remission after the first surgery with histopathological confirmation of an adenoma. A suspicious lesion was seen on the stalk on preoperative MRI. She underwent exploration of the gland via an extended approach. The tuberculum was drilled. Calcified tissue from the previous surgery was seen in the sella and was removed. No adenoma was identified in the sella; thus, the stalk was explored. Dura over the planum was cut and normal pituitary stalk was seen. Adenoma was identified on the stalk and removed. Closure was performed using fat and fascia as there was CSF leak intraop. Postop the patient underwent remission, and histopathology revealed the presence of an adenoma. She developed recurrence a year later and underwent bilateral adrenalectomy for the same.

### 9:16 Algorithmic Management of MRI–Negative Cushing’s Disease.

Based on our experience, we propose the algorithmic management of MRI–Negative Cushing’s disease. In patients with a negative dynamic contrast-enhanced MRI of the sella with central localization on IPSS, imaging using gallium DOTANOC PET-CT can be used to rule out an alternative source of ACTH secretion. After ruling out the differential diagnosis of ectopic Cushing’s disease, a 3T pituitary perfusion MRI should be performed as an adjunct to localize an adenoma. If an adenoma is identified on these, the patient should undergo exploration via a standard endoscopic endonasal transsphenoidal approach. If an adenoma is not identified on these, exploration of the pituitary gland should be performed via an extended endoscopic endonasal transsphenoidal approach. In case of persistence or recurrence after exploration, repeat imaging should be performed and reexploration should be considered via an extended approach. This surgical exploration is planned after several rounds of tumor board meetings. The gland and the lateral sella should be explored first, followed by exploration of the cavernous sinus or pituitary stalk in case of suspicion of an ectopic tumor in these sites. If still no adenoma is identified intraop, a hemihypophysectomy should be performed. In case of nonremission after reexplorations, the patient should undergo further management with bilateral adrenalectomy or stereotactic radiosurgery or medical management. Thank you.

## Acknowledgments

We would like to thank the technical and application specialists from the Neurosurgery Skills Training Facility, Neurosurgery Education and Training School, All India Institute of Medical Sciences, New Delhi, India. We thank Mr. Shashi Shekhar and Mr. Trivender Yadav for their valuable support.

## Disclosures

Dr. Sadhwani reported a grant (no. BT/PR13455/CoE/34/24/2015) from the Department of Biotechnology, Ministry of Science and Technology, Government of India. Professor Suri reported being a principal investigator during the conduct of the study.

## Author Contributions

Primary surgeon: Suri. Assistant surgeon: Raheja, Bora. Editing and drafting the video and abstract: Suri, Sadhwani, Raheja, Bora, Garg, R Sharma. Critically revising the work: Suri, Sadhwani, Raheja, Bora, Garg. Reviewed submitted version of the work: Sadhwani, Raheja, Bora, Khadgawat, Garg, R Sharma. Approved the final version of the work on behalf of all authors: Suri. Supervision: Sadhwani, Bora, Khadgawat, Garg. Diagnosis: MC Sharma.
